# Grey matter volume differences across Parkinson’s disease motor subtypes in the supplementary motor cortex

**DOI:** 10.1016/j.nicl.2024.103724

**Published:** 2024-12-10

**Authors:** A. Martin, J. Nassif, L. Chaluvadi, C. Schammel, R. Newman-Norlund, S. Bollmann, J. Absher

**Affiliations:** a College of Arts and Sciences, University of South Carolina, Columbia, SC, USA; b Darla Moore School of Business, University of South Carolina, Columbia, SC, USA; c Arnold School of Public Health, University of South Carolina, Columbia, SC, USA; d Pathology Associates, Greenville, SC, USA; e Department of Communication Sciences and Disorders, University of South Carolina, Columbia, SC, USA; f Centre for Advanced Imaging, The University of Queensland, Brisbane, Australia; g Division of Neurology, Department of Medicine, Prisma Health-Upstate, Greenville, SC, USA; h School of Health Research, Clemson University, Clemson, SC, USA; i Department of Health Sciences, University of South Carolina School of Medicine Greenville, Greenville, SC, USA

**Keywords:** Parkinson’s disease, Neuroimaging, Tremor dominant, Postural instability and gait difficulty, Akinetic rigid

## Abstract

Parkinson’s Disease (PD) is the second most prevalent neurodegenerative disease worldwide due to loss of dopaminergic neurons projecting from the basal ganglia (BG). It is associated with various motor symptoms that are grouped into subtypes, each with different clinical presentations and disease progressions. Neuroimaging biomarkers focusing on regions a part of motor circuits projecting from the BG can distinguish and improve overall subtyping. The supplementary motor cortex (SMC) is well established in PD neuropathology and associated with freezing of gait and bradykinesia, but has not been thoroughly evaluated across subtypes. This study aims to identify volumetric differences of the SMC based on PD subtypes of tremor dominant (TD), postural instability with gait difficulty (PIGD), and akinetic rigid (AR) using data from Parkinson’s Progression Markers Initiative. To segment grey matter volume and extract region of interest values, voxel-based processing was used. Multi-factor ANCOVAs, Tukey Honest Significance Test, and Kruskal-Wallis were utilized for volumetric analyses (α < 0.05). Subjects were classified and evaluated using TD, PIGD, and AR subtypes from the MDS-UPDRS rating scales. Inter-subtype differences in SMC GMV between TD and PIGD were significant in the right hemisphere for females (p = 0.01). No significant inter-subtype differences were found in the TD/AR system. These results support the use of broader motor networks, specifically the SMC in further understanding the neuropathological heterogeneity of PD. Furthermore, it reveals SMC differences across sexes, subtypes, and subtyping systems, calling for further evaluation of subtyping schemas, specifically regarding sex differences.

## Highlights

• Neuroimaging biomarkers can differentiate PD subtypes and improve treatments.
• The supplementary motor cortex is related to PD symptomology like freezing of gait.
• SMC volume differs in females between the TD and PIGD subtypes.
• In PD vs HC men and women show differences in SMC volume across sex and subtypes.
• PD subtypes may need to be reevaluated to include sex and biomarkers.

## Graphical abstract

**Figure:**
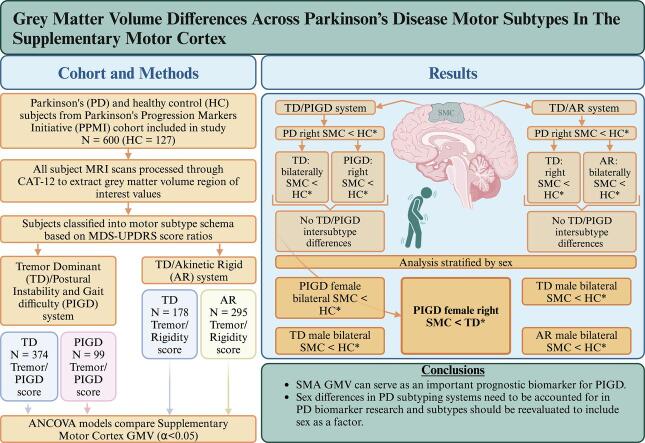

## 1 Introduction

Parkinson’s Disease (PD) is the second most prevalent neurodegenerative disease (Pringsheim et al., 2014) affecting 2–3 % (Willis et al., 2022) of the population ≥ 65 years of age (Poewe et al., 2017). There is a wide variety of motor symptoms associated with PD that present with clinical heterogeneity and disease progression. This has led to the classification of PD into various motor subtypes (Jankovic et al., 1990). While there is not an exact consensus on how to subtype PD (Mestre et al., 2018), the most widely used subtypes include Tremor Dominant (TD), Postural Instability and Gait Difficulty (PIGD), and Akinetic Rigid (AR); (Zetusky et al., 1985, Poewe et al., 2017, Adams et al., 2023) subtypes are determined by self-reported, clinician-verified rating scale ratios via the Movement Disorder Society Unified Parkinson’s Disease Rating Scale (MDS-UPDRS) (Stebbins et al., 2013). As PD advances, TD patients generally show a slower progression in comparison to PIGD patients; the PIGD subtype may also display malignant PD defined as a rapid onset associated with enhanced occupational and physical disability, motor control decline, and akinesia (Jankovic et al., 1990). AR subjects are generally older at onset (Rajput et al., 2009) and exhibit symptoms including rigidity, bradykinesia, and akinesia (Kann et al., 2020). As each subtype has been found to correlate with a unique underlying pathology (Foltynie et al., 2002, Boonstra et al., 2020) and disease progression pattern (Poewe et al., 2017), proper subtype recognition is crucial for appropriate prognosis and treatment (Rajput et al., 2009) to promote optimal patient care and outcomes. Specifically, the identification of pathoanatomical patterns through structural imaging, such as magnetic resonance imaging (MRI), has led to a deeper understanding of the mechanisms behind subtype heterogeneity (Sieber et al., 2014) with the hope of earlier recognition, improved biomarker-based subtyping schemas (Mestre et al., 2021), and improved management through targeted treatments. Biomarkers can be explored by leveraging large repository datasets, such as the Parkinson’s Progression Markers Initiative (PPMI), with vast metadata that increases the power of findings (Simuni et al., 2016).

Efforts have been made to determine subtype characteristics utilizing multi-modal neuroimaging methods such as PET, single-photon emission CT (SPECT), and novel MRI techniques that identify diagnostic imaging biomarkers of each subtype (Marek et al., 2018, Boonstra et al., 2020), such as differences in global and regional gray matter volume (GMV) (Rosenberg-Katz et al., 2013, Rosenberg-Katz et al., 2016, Poewe et al., 2017, Boonstra et al., 2020). TD subjects have displayed less gray matter atrophy in the frontal, parietal, occipital, and temporal lobes as well as the caudate nucleus and cerebellum culmen; TD patients also exhibit larger gray matter volumes in the amygdala and globus pallidus compared to PIGD patients, with no cerebellar differences (Rosenberg-Katz et al., 2016). AR patients have lower GMV in the paracentral (PCL) and posterior superior parietal lobe (SPL), regions that involve the planning and execution of movements (Kann et al., 2020). Among the three motor subtype classifications, non-tremor-dominant patients obtain more severe motor scores than patients with TD (Ren et al., 2020). The extent to which advancing age or disease duration impacts the pattern of these distinctions is unclear (Herb et al., 2016).

Subtyping by neuroimaging is currently limited due to the lack of correlative data and the complexity of clinical symptoms and disease progression, which confound absolute typing (Cao et al., 2022). Furthermore, neuroimaging studies that focus on subtyping have relied on single institutional data or used smaller sample sizes from repositories (Rosenberg-Katz et al., 2013, Pereira et al., 2014 3;82(22):2017–25., Kang et al., 2015, Gu et al., 2016, Li et al., 2017, Uribe et al., 2018, Guo et al., 2022), limiting the utility of these studies in establishing the comprehensive data needed, therefore demonstrating the need for larger scale analyses.

Whilst the basal ganglia (BG) and substantia nigra (SNc) historically are the primary anatomical regions investigated in PD, this parochial view overlooks contributions of the cortex and cerebellum that connect to the BG through motor circuits (Rahimpour et al., 2022). One crucial cortical region, the supplementary motor cortex (SMC), receives the majority of its neuronal input from the BG (Eckert et al., 2006, Wu et al., 2009, Esposito et al., 2013) and is involved in multiple motor circuits projecting from the BG, including a hyperdirect pathway (Oswal et al., 2020; Rahimpour et al., 2022). The SMC plays an essential role in movement planning, initiation, and lower body control (Akkal et al., 2007, Nachev et al., 2008) and has been implicated in PD symptoms like freezing of gait (FOG), bradykinesia, and akinesia (Brugger et al., 2020, Oswal et al., 2021, Li et al., 2022, Rahimpour et al., 2022 Jan). However, despite evidence linking the loss of dopaminergic neurons in the SMC to cardinal PD symptoms and reduced SMC volume in PD patients; (Jahanshahi et al., 1995, Akkal et al., 2007, Wu et al., 2012) it has not been thoroughly evaluated in PD motor subtypes. It has been suggested that evaluation of the SMC and closely related components of the extrapyramidal motor system by quantitative neuroimaging methods may assist in further defining subtypes of PD (Wu et al., 2012, Boonstra et al., 2020, Cao et al., 2022).

Therefore, the purpose of this study is to utilize the publicly available PPMI [www.ppmi-info.org] aggregate database, including MDS-UPDRS data and MRI scans, to determine if GMV in the SMC and closely related regions differentiate TD, PIGD, AR, and Healthy Controls (HC).

**These comparisons aim to test two primary hypotheses: First, that SMC volume will be smaller in PIGD than TD. Secondly, SMC patterns in the TD/PIGD system will differ from those of the TD/AR system, showing that TD/PIGD may be of better use when utilizing the SMC as a biomarker.**

## 2 Methods

### 2.1 Subjects

The Image and Data Archive [ida.loni.usc.edu] was used to query the Parkinson’s Progression Markers Initiative (PPMI) Database, data used was classified as PPMI tier I publicly accessible data. All demographic and clinical data including MDS-UPRS, and MRI data for all PD and HC subjects in the database were downloaded on 03/12/2023. Subjects who did not have MDS-UPDRS data were excluded. Those without GMV data were excluded. Subjects classified as members of the subjects without evidence of dopaminergic deficit (SWEDD) and “Prodromal” (participants at risk of Parkinson’s based on clinical features, genetic variants, or other biomarkers) cohorts were also excluded from the analysis. Therefore, we included only non-SWEDD PD subjects with MDS-UPDRS scores suitable for characterizing their PD motor subtypes, who also had a structural MRI scan amenable to analysis for inter-group, cross-sectional comparisons of GMV differences by ROI. HC subjects were determined by their cohort definition in the PPMI database.

### 2.2 MRI image processing

All data was uploaded to an Oracle Cloud Infrastructure environment configured with Neurodesk (v20230531; Renton et al., 2024). All subject files were then processed through the Computational Anatomy Toolbox (CAT12; Statistical Parameter Mapping (SPM12) software, Wellcome Department of Cognitive Neurology, University College London, London UK) embedded in Neurodesk to normalize each subject's MRI into stereotaxic space and align them with the Neuromorphometrics atlas (Neuromorphometrics Inc., Somerville, MA).

Voxel-Based processing, provided by CAT12, was used to segment and export region of interest (ROI) values of gray matter volume (GMV), white matter volume (WMV), and total intracranial volume (TIV). ROI GMV measurements were normalized as a fraction of TIV by dividing each ROI’s GMV by TIV to account for the correlation between cortical GMV and TIV.

### 2.3 Subtype classifications

Due to the differences in classification between subtyping systems, the motor symptoms scores and ratios were calculated separately to determine a given subject’s motor subtype in both the TD/PIGD system and TD/AR system.

In order to differentiate PD subtypes using the TD/PIGD system, a ratio of mean tremor symptom score (TSS; MDS-UPDRS items 2.10, 3.15a, 3.15b, 3.16a, 3.16b, 3.17a, 3.17b, 3.17c, 3.17d, 3.17e, and 3.18) to mean gait symptom score (GDSS; MDS-UPDRS items 2.12, 2.13, 3.10, 3.11, and 3.12) was calculated for each subject, using the MDS-UPDRS responses from the PPMI database (Stebbins et al., 2013). Subjects with a ratio ≥ 1.15 were classified as the TD subtype and those with a ratio ≤ 0.90 were classified as PIGD (Stebbins et al., 2013). Indeterminate subjects (those with a ratio > 0.90 and < 1.15) were excluded (Stebbins et al., 2013).

To classify subjects under the TD/AR system, a ratio of mean TSS (MDS-UPDRS items 2.10, 3.15a, 3.15b, 3.16a, 3.16b, 3.17a, 3.17b, 3.17c, 3.17d, 3.17e, and 3.18) to mean rigidity symptoms (RSS; MDS-UPDRS items 3.2, 3.3a, 3.3b, 3.3c, 3.3d, 3.3e, 3.4a, 3.4b, 3.6a, 3.6b, 3.7a, 3.7b, 3.8a, 3.8b, 3.14) was calculated for each subject, using MDS-UPDRS responses from the PPMI database (Adams et al., 2023). Those with a ratio ≤ 0.71 were classified as AR, and those with a ratio ≥ 0.82 were classified as TD (Adams et al., 2023). Those with a ratio > 0.71 and < 0.82 were classified as indeterminate and excluded (Adams et al., 2023).

### 2.4 Statistical analysis

PD subjects were age-, sex-, and race-matched to HC subjects using the “MatchIt” (Ho et al., 2011) function in R with Euclidean distance, nearest method and replacement. To track the overlap between subtyping methods, subjects were also assigned a combined subtype with their classification under the TD/PIGD system first and their classification under the TD/AR system denoted second (TD/TD, TD/AR, PIGD/TD, PIGD/AR, HC/HC) (Nassif et al., 2024). Ten outliers (2 TD/TD, 1 TD/AR, and 7 HC/HC), determined by the interquartile range of age were excluded.

Because the cortical thickness of the Anterior Cingulate Gyrus (ACgG) and GMV in the Caudate Nucleus (CN) have been observed to vary across motor subtypes, normalized ROI GMV for the ACgG and CN were also utilized as covariate data along with a few other regions commonly implicated in PD pathology in association with the SMC: the Pallidum, Putamen, Thalamus, Amygdala, and the Accumbens area (Rosenberg-Katz et al., 2016, Chen et al., 2022, Chen et al., 2022, Boonstra et al., 2020, He et al., 2020). Utilizing the baseline MDS-UPDRS date and recorded date of diagnosis, PD duration was calculated in days. Treatment status as a binary variable indicating a “Yes” or “No” response to receipt of PD treatment (medication or deep brain stimulation) was also collected from MDS-UPDRS surveys. To compare descriptive statistics of the motor subtype groups, two sample t-tests were conducted for continuous descriptors and chi-squared analyses were conducted for categorical descriptors.

A multi-factor ANCOVA with covariates for age, sex, race, mean MDS-UPDRS I-III score, days since PD diagnosis, PD treatment status, and chosen ROIs was utilized to compare Left and Right SMC GMV across subjects and isolate statistically significant variance across TD, PIGD, and HC groups as well as across TD, AR, and HC groups. Similar ANCOVAs were run by sex to isolate the variation in SMC GMV between subtypes for each sex. A multiple comparisons Tukey Honest Significance Test was run to isolate mean SMC GMV differences between groups where the ANCOVA demonstrated statistically significant variation in SMC GMV across subtypes. Because some distributions of SMC GMV were bimodal for PIGD and AR subjects, Kruskal-Wallis tests were also run. Given the number of comparisons required to compare subtypes across sex and hemisphere, the Benjamini-Hochberg procedure was implemented with the “P.Adjust” R function to control the false discovery rate (Benjamini and Hochberg, 1995). Corrections were applied separately to each sub-analysis. For the sub-analyses in Table 1, Table 2, nine p-values were used in each correction. For Table 3, Table 4, six p-values were used in separate correction procedures for each variable in the ANCOVA models. After correction, the alpha value of 0.05 (α = 0.05) was considered significant.

**Table 1:** Characteristics of TD vs. PIGD subjects.

	**Overall (N = 600)**	**HC (N = 127)**	**TD** **(N = 374)**	**PIGD** **(N = 99)**	**p**
**Age**					0.618
**Mean (SD)**	63.1 (9.21)	63.1 (9.44)	63.0 (9.15)	63.8 (9.19)	
**Median [Min, Max]**	63.9 [36.6, 84.9]	63.7 [40.2, 82.7]	63.6 [36.6, 84.9]	65.2 [38.5, 82.3]	
**Sex**					0.907
**Female**	221 (36.8 %)	49 (38.6 %)	135 (36.1 %)	37 (37.4 %)	
**Male**	379 (63.2 %)	78 (61.4 %)	239 (63.9 %)	62 (62.6 %)	
**Race**					0.907
**American Indian/Alaska Native**	1 (0.2 %)	0 (0 %)	1 (0.3 %)	0 (0 %)	
**Asian**	9 (1.5 %)	1 (0.8 %)	7 (1.9 %)	1 (1.0 %)	
**Black**	8 (1.3 %)	1 (0.8 %)	6 (1.6 %)	1 (1.0 %)	
**Not Specified**	1 (0.2 %)	0 (0 %)	1 (0.3 %)	0 (0 %)	
**Unknown**	3 (0.5 %)	1 (0.8 %)	1 (0.3 %)	1 (1.0 %)	
**White**	578 (96.3 %)	124 (97.6 %)	358 (95.7 %)	96 (97.0 %)	
**TIV**					0.388
**Mean (SD)**	1510 (153)	1480 (156)	1520 (150)	1500 (156)	
**Median [Min, Max]**	1510 [1070, 1980]	1470 [1160, 1830]	1520 [1070, 1980]	1510 [1150, 1900]	
**Left SMC GMV**					0.618
**Mean (SD)**	4.38 (0.693)	4.39 (0.670)	4.39 (0.707)	4.33 (0.671)	
**Median [Min, Max]**	4.34 [2.14, 6.56]	4.37 [3.17, 6.56]	4.35 [2.14, 6.35]	4.29 [3.00, 6.32]	
**Right SMC GMV**					0.733
**Mean (SD)**	4.20 (0.665)	4.24 (0.672)	4.20 (0.653)	4.16 (0.702)	
**Median [Min, Max]**	4.17 [1.82, 6.73]	4.19 [2.89, 6.12]	4.18 [1.82, 6.11]	4.11 [2.92, 6.73]	
**Days from PD Diagnosis**					**0.00192**
**Mean (SD)**	281 (419)	0 (0)	310 (382)	531 (594)	
**Median [Min, Max]**	122 [0, 2560]	0 [0, 0]	153 [0, 2560]	273 [31.0, 2470]	
**PD Treatment**					**< 0.001**
**0**	525 (87.5 %)	127 (100 %)	334 (89.3 %)	64 (64.6 %)	
**1**	75 (12.5 %)	0 (0 %)	40 (10.7 %)	35 (35.4 %)	
**Tremor Symptom Score**					**< 0.001**
**Mean (SD)**	0.432 (0.377)	0.0336 (0.0984)	0.631 (0.318)	0.187 (0.233)	
**Median [Min, Max]**	0.364 [0, 1.91]	0 [0, 0.636]	0.545 [0.0909, 1.91]	0.0909 [0, 1.27]	
**Gait Difficulty Symptom Score**					**< 0.001**
**Mean (SD)**	0.206 (0.273)	0.0110 (0.0769)	0.170 (0.172)	0.596 (0.359)	
**Median [Min, Max]**	0.200 [0, 2.20]	0 [0, 0.800]	0.200 [0, 1.00]	0.400 [0.200, 2.20]	
**Rigidity Symptom Score**					**0.0160**
**Mean (SD)**	0.715 (0.544)	0.0346 (0.0717)	0.868 (0.462)	1.01 (0.463)	
**Median [Min, Max]**	0.667 [0, 2.27]	0 [0, 0.467]	0.800 [0.0667, 2.27]	0.933 [0.133, 2.07]	
**Mean MDS-UPDRS I-III Score**					**0.0236**
**Mean (SD)**	0.462 (0.318)	0.0373 (0.0495)	0.559 (0.247)	0.639 (0.284)	
**Median [Min, Max]**	0.462 [0, 1.83]	0.0192 [0, 0.250]	0.519 [0.0769, 1.58]	0.635 [0.0769, 1.83]	

*p--values represent the comparison of TD and PIGD subjects using a *t*-test for continuous variables and a Chi-Squared test for factors. HC subjects are included for reference. Supplementary motor area GMV is in cm^3^. PD Treatment denotes whether DBS or medication was received by the patient (0 indicates“NO”; 1 indicates “YES”). Bolded p-values are significant at p < 0.05 after Benjamini-Hochberg correction.

**Table 2:** Characteristics of TD vs. AR subjects.

	**Overall** **(N = 611)**	**HC** **(N = 127)**	**TD** **(N = 384)**	**PIGD** **(N = 100)**	**p**
**Age**					0.542
**Mean (SD)**	63.1 (9.24)	63.1 (9.44)	63.0 (9.19)	63.6 (9.23)	
**Median [Min, Max]**	63.9 [36.6, 84.9]	63.7 [40.2, 82.7]	63.6 [36.6, 84.9]	65.0 [38.5, 82.3]	
**Sex**					0.975
**Female**	225 (36.8 %)	49 (38.6 %)	139 (36.2 %)	37 (37.0 %)	
**Male**	386 (63.2 %)	78 (61.4 %)	245 (63.8 %)	63 (63.0 %)	
**Race**					0.838
**American Indian/Alaska Native**	1 (0.2 %)	0 (0 %)	1 (0.3 %)	0 (0 %)	
**Asian**	9 (1.5 %)	1 (0.8 %)	7 (1.8 %)	1 (1.0 %)	
**Black**	8 (1.3 %)	1 (0.8 %)	6 (1.6 %)	1 (1.0 %)	
**Not Specified**	1 (0.2 %)	0 (0 %)	1 (0.3 %)	0 (0 %)	
**Unknown**	3 (0.5 %)	1 (0.8 %)	1 (0.3 %)	1 (1.0 %)	
**White**	589 (96.4 %)	124 (97.6 %)	368 (95.8 %)	97 (97.0 %)	
**TIV**					0.259
**Mean (SD)**	1510 (154)	1480 (156)	1520 (151)	1500 (160)	
**Median [Min, Max]**	1510 [1070, 1980]	1470 [1160, 1830]	1520 [1070, 1980]	1510 [1150, 1900]	
**Left SMC GMV**					0.416
**Mean (SD)**	4.38 (0.696)	4.39 (0.670)	4.39 (0.713)	4.33 (0.668)	
**Median [Min, Max]**	4.34 [2.14, 6.56]	4.37 [3.17, 6.56]	4.35 [2.14, 6.35]	4.30 [3.00, 6.32]	
**Right SMC GMV**					0.618
**Mean (SD)**	4.21 (0.667)	4.24 (0.672)	4.21 (0.657)	4.17 (0.701)	
**Median [Min, Max]**	4.17 [1.82, 6.73]	4.19 [2.89, 6.12]	4.18 [1.82, 6.11]	4.12 [2.92, 6.73]	
**Tremor Symptom Score**					**< 0.001**
**Mean (SD)**	0.441 (0.387)	0.0336 (0.0984)	0.642 (0.329)	0.185 (0.232)	
**Median [Min, Max]**	0.455 [0, 2.00]	0 [0, 0.636]	0.636 [0.0909, 2.00]	0.0909 [0, 1.27]	
**PIGD Symptom Score**					**< 0.001**
**Mean (SD)**	0.209 (0.276)	0.0110 (0.0769)	0.174 (0.182)	0.592 (0.359)	
**Median [Min, Max]**	0.200 [0, 2.20]	0 [0, 0.800]	0.200 [0, 1.40]	0.400 [0.200, 2.20]	
**Rigidity Symptom Score**					**0.005**
**Mean (SD)**	0.720 (0.544)	0.0346 (0.0717)	0.869 (0.462)	1.02 (0.463)	
**Median [Min, Max]**	0.667 [0, 2.27]	0 [0, 0.467]	0.800 [0.0667, 2.27]	0.967 [0.133, 2.07]	

*p-values represent the comparison of TD and AR subjects using a *t*-test for continuous variables and a Chi-Squared test for factors; HC subjects are included for reference. Supplementary motor area GMV is in cm^3^. PD Treatment denotes whether DBS or medication was received by the patient (0 indicates “NO”; 1 indicates “YES”). Bolded p-values are significant at p < 0.05 after Benjamini-Hochberg correction.

**Table 3:** Comparison of TD, PIGD, and HC utilizing ANCOVA and Kruskal-Wallis.

	**F-Value**	**p-value**	Kruskal-Wallis p-value*
**Overall Left SMC GMV**			
**Subtypes (TD vs. PIGD vs. HC)**	3.000	0.0606	0.134
**Age**	58.747	**< 0.001**	
**Sex**	10.601	**0.00120**	
**Race**	2.277	0.105	
**Mean MDS I-III**	2.185	0.210	
**Days from PD Diagnosis**	5.018	0.146	
**PD Treatment**	0.078	0.780	
**Left Caudate Nucleus**	62.541	**< 0.001**	
**Left Anterior Cingulate Gyrus**	76.525	**< 0.001**	
**Left Pallidum**	0.712	0.479	
**Left Putamen**	4.021	0.0789	
**Left Thalamus Proper**	25.232	**< 0.001**	
**Left Amygdala**	4.118	0.129	
**Left Accumbens Area**	0.542	0.950	
**Overall Right SMC GMV**			
**Subtypes (TD vs. PIGD vs. HC)**	5.205	**0.0172**	0.0734
**Age**	64.309	**< 0.001**	
**Sex**	11.260	**0.00120**	
**Race**	1.959	0.124	
**Mean MDS I-III**	2.975	0.210	
**Days from PD Diagnosis**	3.894	0.146	
**PD Treatment**	0.608	0.651	
**Right Caudate Nucleus**	78.220	**< 0.001**	
**Right Anterior Cingulate Gyrus**	32.765	**< 0.001**	
**Right Pallidum**	1.217	0.406	
**Right Putamen**	13.636	**0.00146**	
**Right Thalamus Proper**	15.237	**< 0.001**	
**Right Amygdala**	1.765	0.369	
**Right Accumbens Area**	0.004	0.950	
**Female Left SMC GMV**			
**Subtypes (TD vs. PIGD vs. HC)**	1.135	0.323	0.537
**Age**	35.605	**< 0.001**	
**Race**	3.039	0.105	
**Mean MDS I-III**	2.561	0.210	
**Days from PD Diagnosis**	2.892	0.146	
**PD Treatment**	4.142	0.259	
**Left Caudate Nucleus**	31.200	**< 0.001**	
**Left Anterior Cingulate Gyrus**	34.332	**< 0.001**	
**Left Pallidum**	0.187	0.666	
**Left Putamen**	0.779	0.378	
**Left Thalamus Proper**	32.338	**< 0.001**	
**Left Amygdala**	0.788	0.456	
**Left Accumbens Area**	2.661	0.626	
**Female Right SMC GMV**			
**Subtypes (TD vs. PIGD vs. HC)**	4.761	**0.0191**	0.0602
**Age**	45.698	**< 0.001**	
**Race**	2.614	0.105	
**Mean MDS I-III**	3.191	0.210	
**Days from PD Diagnosis**	1.975	0.161	
**PD Treatment**	1.752	0.561	
**Right Caudate Nucleus**	24.276	**< 0.001**	
**Right Anterior Cingulate Gyrus**	33.733	**< 0.001**	
**Right Pallidum**	3.370	0.204	
**Right Putamen**	3.619	0.0789	
**Right Thalamus Proper**	12.649	**< 0.001**	
**Right Amygdala**	0.000	0.991	
**Right Accumbens Area**	0.055	0.950	
**Male Left SMC GMV**			
**Subtypes (TD vs. PIGD vs. HC)**	4.055	**0.0272**	0.0602
**Age**	26.868	**< 0.001**	
**Race**	1.437	0.252	
**Mean MDS I-III**	0.760	0.384	
**Days from PD Diagnosis**	2.570	0.146	
**PD Treatment**	0.379	0.651	
**Left Caudate Nucleus**	33.096	**< 0.001**	
**Left Anterior Cingulate Gyrus**	40.475	**< 0.001**	
**Left Pallidum**	1.261	0.406	
**Left Putamen**	3.407	0.0789	
**Left Thalamus Proper**	4.126	**< 0.001**	
**Left Amygdala**	8.574	**0.0218**	
**Left Accumbens Area**	0.185	0.950	
**Male Right SMC GMV**			
**Subtypes (TD vs. PIGD vs. HC)**	6.841	**0.00728**	0.0519
**Age**	24.544	**< 0.001**	
**Race**	1.177	0.320	
**Mean MDS I-III**	0.950	0.384	
**Days from PD Diagnosis**	2.407	0.146	
**PD Treatment**	0.372	0.651	
**Right Caudate Nucleus**	52.321	**< 0.001**	
**Right Anterior Cingulate Gyrus**	8.540	**< 0.001**	
**Right Pallidum**	4.417	0.204	
**Right Putamen**	10.162	**0.00468**	
**Right Thalamus Proper**	3.350	**< 0.001**	
**Right Amygdala**	0.774	0.456	
**Right Accumbens Area**	0.090	0.950	

TD, PIGD, and HC groups were compared; column 'p' reports the ANCOVA p-value. Bolded p-values are significant at alpha = 0.05 after adjustment with the Benjamini-Hochberg method.

**Table 4:** Tukey Differences in mean normalized SMC GMV.

	**TD vs. HC**	**PIGD vs. HC**	**TD vs. PIGD**
**Overall Right SMC GMV**	−0.275 [-0.481, −0.0696] (**p = 0.00495**)	−0.273 [-0.541, −0.00444] (**p = 0.0453**)	−0.00239 [-0.229, 0.224] (p = 0.999)
**Female Right SMC GMV**	−0.0538 [-0.365, 0.257] (p = 0.912)	−0.475 [-0.881, −0.0687] (**p = 0.0173**)	0.421 [0.0750, 0.768] (**p = 0.0125**)
**Male Left SMC GMV**	−0.322 [-0.588, −0.0554] (**p = 0.0131**)	−0.223 [-0.571, 0.124] (p = 0.286)	−0.0984 [-0.390, 0.193] (p = 0.706)
**Male Right SMC GMV**	−0.396 [-0.664, −0.129] (**p = 0.00158**)	−0.150 [-0.499, 0.199] (p = 0.570)	−0.246 [-0.538, 0.0460] (p = 0.118)

This table reports the results of Tukey family wise two way t-tests for the ANCOVA analyses that yielded significant results for variance in mean SMC GMV as a fraction of TIV across motor subtypes. Reported values are the difference in z scores of mean normalized SMC GMV between the subtype groups. The confidence interval is in brackets. Bolded p-values are significant.

## 3 Results

### 3.1 All subjects characteristics

**Fig. 1:**
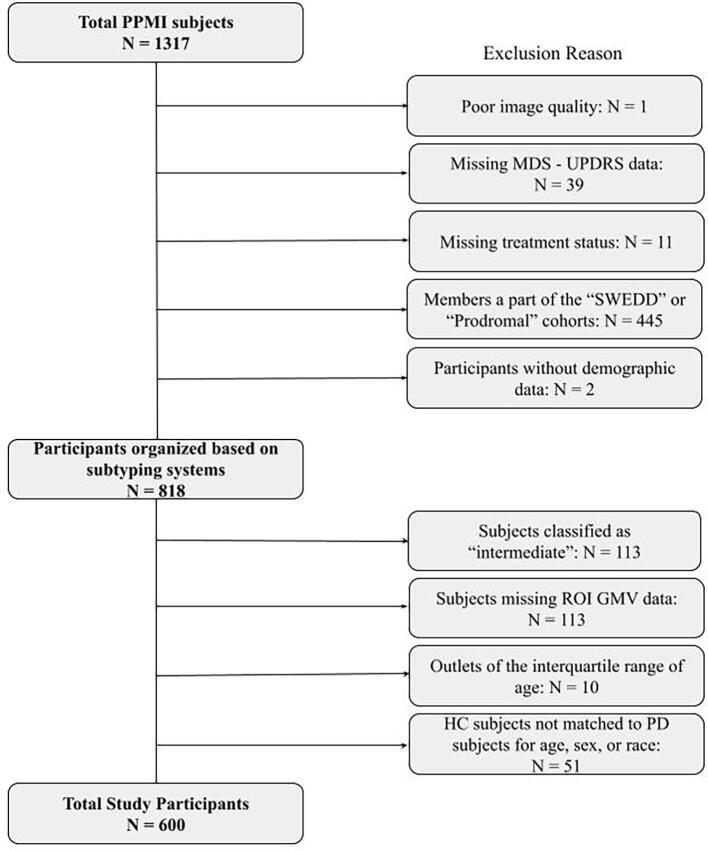
Subject Selection. Inclusion and Exclusion Criteria are outlined.

**Fig. 2:**
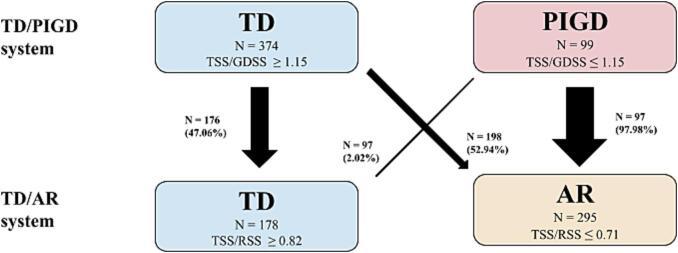
Subtype Schema Switching Diagram. Graphical representation of cross sectional overlap between PD subtyping schemas. Boxes display the motor subtype, number of subjects in each subtype, and classification criteria. Heavier weighted arrows demonstrate higher frequency of subject reclassification.

Overall, 1317 subjects were included in the initial sample. Those without age, sex, GMV, TIV, and MDS-UPDRS data were excluded, and after removing outliers based on age and matching for age, sex, and race, 600 subjects were included in the study (Fig. 1).Fig. 2..

The mean age of the entire cohort was 63.1; 36.8 % (N = 221) were female, and 63.2 % (N = 379; Table 1) were male. Racial composition of the entire cohort was predominantly white (N = 578; 96.3 %; Table 1). The mean TIV for the entire cohort was 1510 cm^3^ (Table 1). The entire cohort had a mean left SMC GMV of 4.38 cm^3^ and a mean GMV of 4.20 cm^3^ (Table 1) for the right SMC. Mean duration since PD diagnosis was 281 days and 75 subjects (12.5 %) had received some form of PD treatment (Table 1). Overall, TSS was 0.432, GDSS was 0.206, and RSS was 0.715 (Table 1). Mean MDS-UPDRS I-III score was 0.462 for the entire cohort (Table 1.).

### 3.2 TD/PIGD subtype characteristics

To compare TD vs. PIGD subjects, data were stratified into two groups: TD (N = 374) and PIGD (N = 99) (HC N = 127 were included for reference; Table 1) using the ratio TSS to GDSS from the subjects’ MDS-UPDRS data. No significant differences in age (p = 0.618), sex (p = 0.907), race (p = 0.907), mean TIV (p = 0.388), mean left SMC GMV (p = 0.618), or mean right SMC GMV (p = 0.733) were observed between TD and PIGD subjects. For TD, 40 subjects (10.7 %) had received PD treatment while 35 of the PIGD subjects (35.4 %; p < 0.001) had received PD treatment. For TD subjects mean TSS was 0.631 and 0.187 (p < 0.001) for PIGD subjects. Mean GDSS was 0.170 for TD subjects and 0.596 (p < 0.001) for PIGD subjects. Mean RSS was 0.868 for TD subjects and 1.01 (p = 0.0160) for PIGD subjects. Mean MDS-UPDRS I-III score was 0.559 for TD and 0.639 for PIGD (p = 0.0236; Table 1).

### 3.3 TD/AR subtype characteristics

To compare reclassified TD vs. AR subjects, data were again stratified into two groups: TD (N = 178) and AR (N = 295) (HC N = 127 were included for reference; Table 2) using the ratio of TSS to RSS from the subjects’ MDS-UPDRS data. No significant differences in age (p = 0.534), sex (p = 0.534), race (p = 0.105), mean TIV (p = 0..105), mean left SMC GMV (p = 0.534), mean right SMC GMV (p = 0.544), or mean duration since PD diagnosis (p = 0.138) were observed between TD and PIGD subjects. TD subjects had a mean TSS of 0.783 and AR subjects had a mean TSS of 0.390 (p < 0.001). TD subjects had a mean GDSS of 0.172 and AR subjects had a mean GDSS of 0.311 (p < 0.001). TD subjects had a mean RSS of 0.586 and AR subjects had a mean RSS of 1.09 (p < 0.001). Mean MDS-UPDRS I-III score was 0.483 for TD and 0.631 for AR (p < 0.001; Table 2).

### 3.4 Contrasting PD subtyping systems

#### 3.4.1 SMC in TD/PIGD and HC comparisons

Variance across subtype differentiation (TD vs. PIGD vs. HC) in the mean SMC GMV as a fraction of TIV was not significant in the left (F = 3.000; p = 0.0606) but was significant in the right hemisphere (F = 5.205; p = 0.0172; Table 3) when subjects were age, sex and race matched and controlled for age, sex, race, CN GMV, ACgG GMV, Pallidum GMV, Putamen GMV, TP GMV, and Accumbens Area GMV as covariates. In both hemispheres, age (Left: p < 0.001; Right: p < 0.001), sex (Left: p = 0.00120; Right: p = 0.00120), CN GMV (Left: p < 0.001; Right: p < 0.001), ACgG GMV (Left: p < 0.001; Right: p < 0.001), and TP GMV (Left: p < 0.001; Right: p < 0.001; Table 3) were significantly associated with variance in SMC GMV. Kruskal-Wallis analysis showed that variance across subtypes in mean SMC GMV was not statistically significant in the left (p = 0.134) or right hemisphere (p = 0.0734; Table 3).

When separated by sex, variance in mean left SMC GMV was not significantly associated with motor subtype (TD vs. PIGD vs. HC) for female subjects (F = 1.135; p = 0.323) but was significant for males (F = 4.055; p = 0.0272; Table 3). Variance in mean SMC GMV in the right hemisphere across motor subtypes was statistically significant in both females (F = 4.761; p = 0.0191) and males (F = 6.841; p = 0.00728; Table 3). For females, age (Left: p < 0.001; Right: p < 0.001), sex (Left: p = 0.00120; Right: p = 0.00120), CN GMV (Left: p < 0.001; Right: p < 0.001), ACgG GMV (Left: p < 0.001; Right: p < 0.001), and TP GMV (Left: p < 0.001; Right: p < 0.001; Table 3) were significantly associated with variance in SMC GMV in both hemispheres. For males, age (Left: p < 0.001; Right: p < 0.001), sex (Left: p = 0.00120; Right: p = 0.00120), CN GMV (Left: p < 0.001; Right: p < 0.001), ACgG GMV (Left: p < 0.001; Right: p < 0.001), and TP GMV (Left: p < 0.001; Right: p < 0.001) were significantly associated with variance in SMC GMV in both hemispheres along with Amygdala GMV in the left hemisphere (p = 0.0218) and Putamen GMV in the right hemisphere (p = 0.00468; Table 3). Kruskal-Wallis analysis showed that variance across subtypes in mean SMC GMV was not statistically significant in either hemisphere for females (Left: p = 0.537; Right: p = 0.0602) or males (Left: p = 0.0602; Right: p = 0.0519; Table 3).

Family-wise t-tests obtained from a Tukey’s Honest Significance Test demonstrate a statistically significant difference in mean normalized GMV in the right SMC of −0.275 standard deviations (p = 0.00495) when comparing the TD and HC and a difference of −0.273 standard deviations (p = 0.0453; Table 4) in the right between PIGD and HC.. For females, there was a significant difference in right hemisphere mean SMC GMV of −0.475 standard deviations (p = 0.0173) between PIGD subjects and HCs and a difference of 0.421 standard deviations (p = 0.0125; Table 4) between TD and PIGD subjects. For males, there was a significant difference in mean SMC GMV of −0.322 standard deviations (p = 0.0131) in the left hemisphere and of −0.396 standard deviations (p = 0.00158; Table 4) in the right hemisphere between TD subjects and HC.

#### 3.4.2 SMC in TD/AR and HC comparisons

Variance across subtype differentiation (TD vs. AR vs. HC) in the mean SMC GMV as a fraction of TIV was not significant in the left hemisphere (F = 3.325; p = 0.0550), but was significant in the right hemisphere (F = 5.648; p = 0.0223; Table 5) when subjects were matched on age, sex, and race and controlled for age, sex, race, CN GMV, ACgG GMV, Pallidum GMV, Putamen GMV, TP GMV, and Accumbens Area GMV as covariates. In both hemispheres, age (Left: p < 0.001; Right: p < 0.001), sex (Left: p = 0.00126; Right: p = 0.00126), CN GMV (Left: p < 0.001; Right: p < 0.001), ACgG GMV (Left: p < 0.001; Right: p < 0.001), and TP GMV (Left: p < 0.001; Right: p < 0.001) along with Putamen GMV (p = 0.00157; Table 5) in only the right hemisphere were significantly associated with variance in SMC GMV. Kruskal-Wallis analysis showed that variance across subtypes in mean SMC GMV was not statistically significant in the either hemisphere (Left: p = 0.1440; Right: p = 0.07623; Table 5).When separated by sex, variance in mean SMC GMV across motor subtypes (TD vs. AR vs. HC) was not statistically significant in the left hemisphere for females (F = 1.222; p = 0.682) but was significant for males (F = 3.763; p = 0.0483; Table 5). In the right hemisphere, variance in mean SMC GMV across motor subtypes was not statistically significant for females (F = 1.600; p = 0.245) but was significant for males (F = 4.836; p = 0.0254; Table 5). For females, age (Left: p < 0.001; Right: p < 0.001), sex (Left: p = 0.00120; Right: p = 0.00120), CN GMV (Left: p < 0.001; Right: p < 0.001), ACgG GMV (Left: p < 0.001; Right: p < 0.001), and TP GMV (Left: p < 0.001; Right: p < 0.001; Table 5) were significantly associated with variance in SMC GMV in both hemispheres. For males, age (Left: p < 0.001; Right: p < 0.001), sex (Left: p = 0.00120; Right: p = 0.00120), CN GMV (Left: p < 0.001; Right: p < 0.001), ACgG GMV (Left: p < 0.001; Right: p < 0.001), and TP GMV (Left: p = 0.00480; Right: p = 0.00822) were significantly associated with variance in SMC GMV in both hemispheres along with Amygdala GMV in the left hemisphere (p = 0.0206) and Putamen GMV in the right hemisphere (p = 0.00595; Table 5) Kruskal-Wallis analysis showed that variance across subtypes in mean SMC GMV was not statistically significant in either hemisphere for females (Left: p = 0.682; Right: p = 0.460) or males (Left: p = 0.0762; Right: p = 0.0762; Table 5).

**Table 5:** Comparison of TD, AR, and HC utilizing ANCOVA and Kruskal-Wallis.

	**F-Value**	**p-value**	Kruskal-Wallis p-value*
**Overall Left SMC GMV**			
**Subtypes (TD vs. AR vs. HC)**	3.325	0.0550	0.1440
**Age**	58.380	**< 0.001**	
**Sex**	10.501	**0.00126**	
**Race**	2.289	0.0990	
**Mean MDS I-III**	1.904	0.252	
**Days from PD Diagnosis**	5.027	0.127	
**PD Treatment**	0.060	0.807	
**Left Caudate Nucleus**	62.623	**< 0.001**	
**Left Anterior Cingulate Gyrus**	76.444	**< 0.001**	
**Left Pallidum**	0.705	0.482	
**Left Putamen**	3.953	0.0791	
**Left Thalamus Proper**	25.371	**< 0.001**	
**Left Amygdala**	4.112	0.129	
**Left Accumbens Area**	0.550	0.947	
**Overall Right SMC GMV**			
**Subtypes (TD vs. AR vs. HC)**	5.648	**0.0223**	0.0762
**Age**	63.760	**< 0.001**	
**Sex**	11.141	**0.00126**	
**Race**	1.985	0.119	
**Mean MDS I-III**	2.463	0.252	
**Days from PD Diagnosis**	4.067	0.127	
**PD Treatment**	0.634	0.699	
**Right Caudate Nucleus**	78.216	**< 0.001**	
**Right Anterior Cingulate Gyrus**	32.693	**< 0.001**	
**Right Pallidum**	1.231	0.401	
**Right Putamen**	13.496	**0.00157**	
**Right Thalamus Proper**	15.169	**< 0.001**	
**Right Amygdala**	1.808	0.358	
**Right Accumbens Area**	0.004	0.947	
**Female Left SMC GMV**			
**Subtypes (TD vs. AR vs. HC)**	1.222	0.297	0.682
**Age**	35.019	**< 0.001**	
**Race**	3.143	0.0990	
**Mean MDS I-III**	2.255	0.252	
**Days from PD Diagnosis**	2.361	0.151	
**PD Treatment**	3.226	0.444	
**Left Caudate Nucleus**	31.175	**< 0.001**	
**Left Anterior Cingulate Gyrus**	34.918	**< 0.001**	
**Left Pallidum**	0.101	0.751	
**Left Putamen**	1.000	0.319	
**Left Thalamus Proper**	31.227	**< 0.001**	
**Left Amygdala**	0.885	0.418	
**Left Accumbens Area**	2.859	0.554	
**Female Right SMC GMV**			
**Subtypes (TD vs. AR vs. HC)**	1.600	0.245	0.460
**Age**	46.356	**< 0.001**	
**Race**	2.657	0.0990	
**Mean MDS I-III**	3.919	0.252	
**Days from PD Diagnosis**	0.949	0.331	
**PD Treatment**	0.406	0.699	
**Right Caudate Nucleus**	25.744	**< 0.001**	
**Right Anterior Cingulate Gyrus**	34.050	**< 0.001**	
**Right Pallidum**	2.867	0.276	
**Right Putamen**	3.796	0.0791	
**Right Thalamus Proper**	11.378	**< 0.001**	
**Right Amygdala**	0.000	0.991	
**Right Accumbens Area**	0.068	0.947	
**Male Left SMC GMV**			
**Subtypes (TD vs. AR vs. HC)**	3.763	**0.0483**	0.0762
**Age**	26.868	**< 0.001**	
**Race**	1.456	0.244	
**Mean MDS I-III**	0.753	0.433	
**Days from PD Diagnosis**	2.939	0.131	
**PD Treatment**	0.303	0.699	
**Left Caudate Nucleus**	33.308	**< 0.001**	
**Left Anterior Cingulate Gyrus**	39.814	**< 0.001**	
**Left Pallidum**	1.249	0.401	
**Left Putamen**	3.400	0.0792	
**Left Thalamus Proper**	4.248	**0.00480**	
**Left Amygdala**	8.672	**0.0206**	
**Left Accumbens Area**	0.179	0.947	
**Male Right SMC GMV**			
**Subtypes (TD vs. AR vs. HC)**	4.836	**0.0254**	0.0762
**Age**	24.493	**< 0.001**	
**Race**	1.208	0.304	
**Mean MDS I-III**	0.616	0.433	
**Days from PD Diagnosis**	3.471	0.127	
**PD Treatment**	0.694	0.699	
**Right Caudate Nucleus**	52.253	**< 0.001**	
**Right Anterior Cingulate Gyrus**	8.397	**< 0.001**	
**Right Pallidum**	4.301	0.233	
**Right Putamen**	9.707	**0.00595**	
**Right Thalamus Proper**	3.037	**0.00822**	
**Right Amygdala**	0.912	0.417	
**Right Accumbens Area**	0.086	0.947	

TD, AR, and HC groups are compared; column 'p' reports the ANCOVA p-value. Bolded p-values are significant at alpha = 0.05 after adjustment with the Benjamini-Hochberg method.

Family-wise t-tests obtained from a Tukey’s Honest Significance Test demonstrate a statistically significant difference in mean normalized right SMC GMV of −0.303 standard deviations (p = 0.00243) when comparing the AR subtype with HC. For males, there was a significant difference in the left SMC of −0.317 standard deviations (p = 0.0378) between TD and HC subjects and a difference of −0.293 standard deviations (p = 0.0334; Table 6) between AR and HC subjects. For males, there was a significant difference in the right SMC of −0.345 standard deviations (p = 0.0220) between TD and HC subjects and of −0.346 standard deviations (0.00966) between AR and HC subjects.

**Table 6:** Tukey Differences in mean normalized SMC GMV.

	**TD vs. HC**	**AR vs. HC**	**TD vs. AR**
**Overall Right SMC GMV**	−0.227 [-0.460, 0.00537] (p = 0.0572)	−0.303 [-0.516, −0.0909] (**p = 0.00243**)	0.0762 [-0.114, 0.266] (p = 0.614)
**Male Left SMC GMV**	−0.317 [-0.620, −0.141] (**p = 0.0378**)	−0.293 [-0.567, −0.0182] (**p = 0.0334**)	−0.0245 [-0.270, 0.220] (p = 0.970)
**Male Right SMC GMV**	−0.345 [-0.651, −0.0402] (**p = 0.0220**)	−0.346 [-0.622, −0.0692] (**p = 0.00966**)	0.000140 [-0.247, 0.247] (p = 0.999)

This table reports the results of Tukey family wise two way t-tests for the ANCOVA analyses that yielded significant results for variance in mean SMC GMV as a fraction of TIV across motor subtypes. Reported values are the difference in z scores of mean normalized SMC GMV between the subtype groups. The confidence interval is in brackets Bolded p-values are significant.

## 4 Discussion

PD manifests with a range of dominant symptoms, progression, and key clinical features, leading to the classification of symptom patterns into subtypes (Foltynie et al., 2002). Differentiating subtypes enables clinicians to predict prognosis, attenuate progression, and provide more targeted treatment (Rajput et al., 2009). However, there is not yet a consensus on the best subtyping system (Fereshtehnejad and Postuma, 2017), the most widely accepted appear to be TD/PIGD (Jankovic et al., 1990, Stebbins et al., 2013) and TD/AR (Rajput et al., 2009, Konno et al., 2018, DiMarzio et al., 2020, Adams et al., 2023).

Identifying motor subtype-specific biomarkers may provide insight into the underlying mechanisms behind the heterogeneity of PD and produce more accurate subtypes overall (Mestre et al., 2018), leading to earlier detection of asymptomatic PD patients, precise prognostication, and optimized treatment strategies (Rajput et al., 2009, Poewe et al., 2017, Boonstra et al., 2020). Previous research indicates that neuroimaging-based biomarkers, particularly those focusing on atrophy patterns within PD-specific networks, are valuable predictors of motor, cognitive, and global PD outcomes (Zeighami et al., 2019).

Specifically, understanding how GMV atrophy patterns and altered connectivity correspond to dominant symptoms and subtypes can lead to more individualized treatment such as subtype-based deep brain stimulation (DBS) and transcranial direct current stimulation (tDCS) on ROIs contributing to a patient's particular symptoms (Sadler et al., 2021). PD GMV research has particularly focused on basal ganglia (BG) regions such as the CN, SNc, Subthalamic Nucleus, AGcG, Globus Pallidus, and Putamen. However, more recent studies have shifted attention to cortical regions involved in larger motor pathways projecting to and/or from the BG, such as the BG-thalamo-cortical motor loop (BGTC) and more broadly the cortical–striatal–thalamic circuit (CTC); which are both well-established in PD pathology (Oswal et al., 2021), particularly to dysfunctional connectivity with the SMC (Playford et al., 1992, Haslinger et al., 2001, Akkal et al., 2007, Duann et al., 2009, Wu et al., 2012, Rahimpour et al., 2022 Jan). SMC hypoactivation plays a role in akinesia (Grafton, 2004), bradykinesia (Jahanshahi et al., 1995), and FOG (Della Sala et al., 2002 Jan, Brugger et al., 2020, Bardakan et al., 2022). This further highlights that SMC disruption, associated with PD symptoms like FOG and rigidity will contribute to decreased SMC volume in PIGD subjects. While some studies have investigated SMC differences between PD motor subtypes, many of these studies havehad limited sample size, relied on institutional data, and did not compare both subtyping systems using the same sample of patients.To the best of our knowledge, this study is one of the largest neuroimaging PD analyses (n = 600) and the first to highlight volumetric sex differences across PD motor subtypes. Prior research generally relied on sample sizes of approximately 100 subjects or less out of both institutional (Rosenberg-Katz et al., 2013, Gu et al., 2016, Zheng et al., 2022) and retrospective repository studies (Pereira et al., 2014 3;82(22):2017–25., Uribe et al., 2018, Guo et al., 2022) with very few studies above 200 participants (Li et al., 2018; Zeighami et al., 2019). even in studies also utilizing the PPMI database. Additionally, our age and sex demographics matched those of other PPMI general population studies (Dahodwala et al., 2009, Deliz et al., 2024; Rusillo et al., 2022).

Our finding of significant GMV differences among groups in the TD/PIGD analysis builds upon prior work. For example, an ROI GMV analysis using the TD/PIGD system found that decreased pre-supplementary motor area volume was associated with increased PIGD symptom severity (Rosenberg-Katz et al., 2016). Another study noted TD and AR showed greater activation in the SMC than PIGD during DBS pulse cycling, possibly indicating that those with PIGD experience more dysfunction in SMA-involved pathways (DiMarzio et al., 2020). Interestingly, there appear to be no publications that use a hypothesis-driven approach to investigate the relationship between PD subtypes and SMC. Previous PD studies involving the SMC utilized data-driven approaches that examined global brain changes rather than focusing on the SMC as a specific ROI (Rosenberg-Katz et al., 2016). This may be due to the relatively small size of the SMC as even a small volume loss can result in broader impairments and disrupt specialized circuits essential for motor planning such as the BGTC (Chen et al., 2018, Matías-Guiu et al., 2018).

We hypothesized that cortical areas important for lower body functioning, such as the SMC, will be smaller in those with PIGD when compared to other subtypes based on functional connectivity dysfunction and greater motor impairment. As expected, PD subjects overall (without sex stratification) showed less SMC volume when compared to HC subjects, particularly on the right side. There is no current literature regarding the lateralization of atrophy patterns in the SMC but some research suggests that atrophy lateralization in closely related regions like the ACgC may be due to predominant symptom lateralization (Chen et al., 2022, Chen et al., 2022). Within the overall TD/PIGD and TD/AR system, the SMC showed no difference bilaterally comparing TD and PIGD (Table 3) or TD and AR to HC (Table 4).

Furthermore, to the best of our knowledge there is no current literature regarding the relationship between subtypes, GMV, and how it differs between sexes; even though imaging and PD presentation sex differences are well documented (Cerri et al., 2019, Dahodwala et al., 2009, Deliz et al., 2024, Haaxma et al., 2007, Yadav et al., 2016). When we stratified each subtyping system by sex, the SMC of PIGD females was smaller compared to HC. In contrast, female TD subjects showed no significant difference when compared to HCs. The primary significant finding in males was that SMC volume was bilaterally smaller in TD than HC. The right hemisphere SMC in females was significantly smaller in volume in the PIGD cohort by 0.0185 % of TIV or approximately 0.28 mL difference of brain volume when compared to TD. Within the TD/AR system, males showed smaller SMC volume bilaterally when comparing both TD and AR to HC but no significant findings were found in females; however, no significant differences were noted between TD and AR cohorts for either sex.

Our findings of reduced SMC volume in females with PIGD align with other studies suggesting distinct sex-specific patterns in PD (Cerri et al., 2019). A recent review identified three levels in which sex influences PD pathophysiology differentially: dopaminergic neurodegeneration, neuroinflammation, and oxidative stress (Cerri et al., 2019). This is thought to be a result of the estrogen decrease that occurs during menopause. Later onset of menopause, and estrogen replacement therapy after menopause show a positive correlation with age of onset and neurodegeneration (Haaxma et al., 2007, Unda et al., 2022). Because men also typically present with symptoms consistent with SMC neurodegeneration such as FOG and rigidity and women present with more tremor, significant intersubtype findings in women related to gait difficulty and SMC morphology could be more distinguishable than in men whose GDSS scores are generally higher (Haaxma et al., 2007) While clinical differences between sexes are well established, the pathophysiological mechanism.s behind PD sex differences are still not fully understood and current studies on the topic are limited (Oltra et al., 2024).

Interestingly, when we reclassified subjects from TD/PIGD system to the TD/AR system a substantial number of subjects shifted subtype. Out of the 374 TD subjects, 52.9 % (n = 198) switched from TD to AR, whereas 98 % (n = 97) of PIGD subjects switched to AR. Both groups' demographics were similar in age, sex, and race (Fig. 2). Conversely, only 2 PIGD subjects switched from PIGD to TD when reclassifying. Both subjects were female with a mean age of 71 compared to our average overall age of 63.1. Considering women display a later onset of PD (Russillo et al., 2022) and faster progression of motor symptoms (Georgiev et al., 2017), this further implicates sex differences in motor subtypes and the structural pathology of PD.

Subjects who switched from TD or PIGD to AR show an RSS that far outweighs the TSS, even though the average tremor numerator varies from 0.496 in the TD to AR group to 0.171 in the PIGD to AR group. This implies that AR as a subtype allows for a greater variety of symptoms with the addition of rigidity, resulting in a much broader range of scores and a more heterogeneous cohort compared to PIGD. This may explain why there were no significant intersubtype findings in the TD/AR group. Along with previous research (Konno et al., 2018), PIGD may simply represent a subset of AR symptoms.

Moreover, the high percentage of subjects who switched during subtype reclassification highlights the deeper issue of the validity of the current PD motor subtyping systems. Both the TD/PIGD and TD/AR systems show approximately a 50 % stability rate over time (Cao et al., 2022, Erro et al., 2019, Kohat et al., 2021). However, this number can be improved by considering the TSS, RSS, and levodopa use. Stability is especially crucial to evaluate when researching correlations with biomarkers and long-term prognostication (Simuni et al., 2016). We believe subtyping schemas need to be specifically reevaluated not only to more accurately represent neuropathophysiology (Mitchell et al., 2021), but to reflect the differences in PD pathology between sexes. Given that PD motor subtypes may fluctuate over time, longitudinal morphometry studies may uncover neuroanatomical correlates of these symptom changes and offer insight into varying susceptibilities to neurodegeneration in specific brain regions contributing to distinct patterns of atrophy progression. Moreover, biomarker studies need to make stratification by sex the standard to account for the distinct sex-specific pathogenic mechanisms present in PD (Cerri et al., 2019).

Future volumetric studies should also incorporate diffusion-based analyses to further investigate how specific symptom scores correlate with SMC volume and motor circuit connectivity; thus, providing a deeper functional understanding of the region’s role in specific PD symptoms. Specifically, longitudinal studies conducted using PPMI to investigate how SMC GMV correlates with subtype and MDS-UPDRS symptom progression over time between sexes. Additionally, by leveraging AI and machine learning techniques, these analyses could enhance subtype identification accuracy (Fang et al., 2020), and enable highly accurate, pre-clinical diagnoses (Prashanth et al., 2016, Peng et al., 2017). As machine learning and the body of PD biomarker literature continue to advance, multi-modal diagnostic applications −- integrating both clinical and imaging data – could be embedded into clinical systems. Ideally, this would allow clinicians to upload a patient’s MRI and receive prognosis, subtype classifications, and proposed treatment based on current biomarker research, longitudinal studies, and patients' individual neuroanatomical patterns. This approach would streamline clinical care, improve differential diagnosis accuracy, enable early detection, and support more tailored treatment plans (Zhang, 2022).

One of the limitations of this study is that, while utilization of a publicly available database allows for larger numbers, the quality of data and measures collected cannot be controlled, and therefore variability may exist of which we are unaware, such as MRI quality and scoring variance. As noted in this study, subtype analysis itself can be considered a limitation as the individual subtyping schemas do not fully encapsulate the variety of symptoms of PD and preferentially focus on physical manifestations. Furthermore, the MDS-UPDRS scores themselves rely on subjective assessments which may introduce additional variability into this analysis. The large sample size of our study attempts to combat MRI signal noise and bias introduced. Furthermore, our exclusion criteria excluded subjects classified as intermediate due to TSS/GDSS ratios of > 0.90 and < 1.15 and TSS/RSS ratios of > 0.71 and < 0.82. This introduced potential selection bias into our cohort by excluding those with MDS-UPDRS scores characterized by an even distribution of tremor and non-tremor symptoms. Thus, our results may be generalizable primarily to PD subjects within distinct MDS-UPDRS score-based subtypes, rather than to all individuals with PD. Another limitation of this study is that the subjects included were overwhelmingly 95.6 % white. While PD has been shown to have a greater prevalence in white individuals compared to other races (Dahodwala et al., 2017), this dataset does show a distinct lack of diversity. The racial homogeneity of the PPMI dataset suggests caution when generalizing the results of our study to non-white populations. Future PPMI analyses should consider comparing the PPMI dataset with others that have more diverse samples or integrating diverse samples into their analysis such as the Global Parkinson’s Genetics Program (Lloyd, 2022). Additionally, some potential limitations coincide with the use of VBM: VBM may not fully detect subtle changes in GMV due to smoothing and signal-to-noise ratios from the original MRI scans. We also did not parcellate the pre-SMA from the SMA-proper reducing the fidelity of subregional impact; however, both are important for the sequencing of movements and motor initiation (Shima and Tanji, 1998).

## 5 Conclusion

GMV biomarkers have the potential to elucidate neural structures contributing to particular PD symptomatologies. The SMC is a crucial piece of the anatomical network implicated in PD as it has a direct pathway to the SNc and is a part of many motor circuits subject to PD pathology and its manifestations, specifically rigidity and freezing of gait. We found that SMC GMV is reduced in PD, varies between TD and PIGD subtypes, and differs between sexes. These findings establish an important role for sex stratification, and motor symptom score specification in future biomarker research and subtyping systems. Future work examining imaging-based biomarkers for PD must implement sex stratification and should consider longitudinal analysis, connectivity, and AI techniques.

## Funding Statement

This research did not receive any specific grant from funding agencies in the public, commercial, or not-for-profit sectors.

## CRediT authorship contribution statement

**A. Martin:** Writing – review & editing, Writing – original draft, Methodology, Investigation, Conceptualization. **J. Nassif:** Writing – review & editing, Writing – original draft, Validation, Software, Methodology, Investigation, Formal analysis, Data curation, Conceptualization. **L. Chaluvadi:** Investigation. **C. Schammel:** Writing – review & editing, Supervision, Project administration. **R. Newman-Norlund:** Resources. **S. Bollmann:** Resources. **J. Absher:** Writing – review & editing, Validation, Supervision, Project administration, Funding acquisition, Conceptualization.

## Declaration of Competing Interest

The authors declare that they have no known competing financial interests or personal relationships that could have appeared to influence the work reported in this paper.

## Data Availability

Data will be made available on request.

## References

[b0005] Adams C., Suescun J., Haque A., Block K., Chandra S., Ellmore T.M., Schiess M.C. (2023). Updated Parkinson's disease motor subtypes classification and correlation to cerebrospinal homovanillic acid and 5-hydroxyindoleacetic acid levels. Clin. Park Relat. Disord..

[b0010] Akkal D., Dum R.P., Strick P.L. (2007). Supplementary motor area and presupplementary motor area: targets of basal ganglia and cerebellar output. J. Neurosci..

[b0015] Bardakan M.M., Fink G.R., Zapparoli L., Bottini G., Paulesu E., Weiss P.H. (2022). Imaging the neural underpinnings of freezing of gait in Parkinson's disease. Neuroimage Clin..

[b0020] Benjamini Y., Hochberg Y. (1995). Controlling the false discovery rate: A practical and powerful approach to multiple testing. J. R. Stat. Soc. Ser. B Stat Methodol..

[b0025] Boonstra J.T., Michielse S., Temel Y., Hoogland G., Jahanshahi A. (2020). Neuroimaging detectable differences between Parkinson's disease motor subtypes: a systematic review. Mov. Disord. Clin. Pract..

[b0030] Brugger F., Wegener R., Walch J., Galovic M., Hägele-Link S., Bohlhalter S., Kägi G. (2020). Altered activation and connectivity of the supplementary motor cortex at motor initiation in Parkinson's disease patients with freezing. Clin. Neurophysiol..

[b0035] Cao K., Pang H., Yu H., Li Y., Guo M., Liu Y., Fan G. (2022). Identifying and validating subtypes of Parkinson's disease based on multimodal MRI data via hierarchical clustering analysis. Front. Hum. Neurosci..

[b0040] Cerri S., Mus L., Blandini F. (2019). Parkinson's Disease in Women and Men: What's the Difference?. J. Parkinsons Dis..

[b0045] Chen J., Jiang X., Wu J., Wu H., Zhou C., Guo T., Bai X., Liu X., Wen J., Cao Z., Gu L., Yang W., Pu J., Guan X., Xu X., Zhang B., Zhang M. (2022). Gray and white matter alterations in different predominant side and type of motor symptom in Parkinson's disease. CNS Neurosci. Ther..

[b0050] Chen G., Zhou B., Zhu H., Kuang W., Bi F., Ai H., Gu Z., Huang X., Lui S., Gong Q. (2018). White matter volume loss in amyotrophic lateral sclerosis: A meta-analysis of voxel-based morphometry studies. Prog. Neuropsychopharmacol. Biol. Psychiatry.

[b0055] Dahodwala N., Siderowf A., Xie M., Noll E., Stern M., Mandell D.S. (2009). Racial differences in the diagnosis of Parkinson's disease. *Mov. Disord.*.

[b0060] Deliz J.R., Tanner C.M., Gonzalez-Latapi P. (2024). Epidemiology of Parkinson's disease: an update. Curr. Neurol. Neurosci. Rep..

[b0065] Della Sala S., Francescani A., Spinnler H. (2002 Jan). Gait apraxia after bilateral supplementary motor area lesion. J. Neurol. Neurosurg. Psychiatry.

[b0070] DiMarzio M., Madhavan R., Joel S., Hancu I., Fiveland E., Prusik J., Gillogly M., Rashid T., MacDonell J., Ashe J., Telkes I., Feustel P., Staudt M.D., Shin D.S., Durphy J., Hwang R., Hanspal E., Pilitsis J.G. (2020). Use of functional magnetic resonance imaging to assess how motor phenotypes of parkinson's disease respond to deep brain stimulation. Neuromodulation.

[b0075] Duann J.R., Ide J.S., Luo X., Li C.S. (2009). Functional connectivity delineates distinct roles of the inferior frontal cortex and presupplementary motor area in stop signal inhibition. J. Neurosci..

[b0080] Eckert T., Peschel T., Heinze H.J., Rotte M. (2006). Increased pre-SMA activation in early PD patients during simple self-initiated hand movements. J. Neurol..

[b0085] Erro R., Picillo M., Amboni M., Savastano R., Scannapieco S., Cuoco S., Santangelo G., Vitale C., Pellecchia M.T., Barone P. (2019). Comparing postural instability and gait disorder and akinetic-rigid subtyping of Parkinson disease and their stability over time. Eur. J. Neurol..

[b0090] Esposito F., Tessitore A., Giordano A., De Micco R., Paccone A., Conforti R., Pignataro G., Annunziato L., Tedeschi G. (2013). Rhythm-specific modulation of the sensorimotor network in drug-naive patients with Parkinson's disease by levodopa. Brain.

[b0095] Fang E., Ann C.N., Maréchal B., Lim J.X., Tan S.Y.Z., Li H., Gan J., Tan E.K., Chan L.L. (2020). Differentiating Parkinson's disease motor subtypes using automated volume-based morphometry incorporating white matter and deep gray nuclear lesion load. J. Magn. Reson. Imaging.

[b0100] Fereshtehnejad S.M., Postuma R.B. (2017). Subtypes of Parkinson's Disease: What Do They Tell Us About Disease Progression?. Curr. Neurol. Neurosci. Rep..

[b0105] Foltynie T., Brayne C., Barker R.A. (2002). The heterogeneity of idiopathic Parkinson's disease. J. Neurol..

[b0110] Georgiev D., Hamberg K., Hariz M., Forsgren L., Hariz G.M. (2017). Gender differences in Parkinson's disease: a clinical perspective. Acta Neurol. Scand..

[b0115] Grafton S.T. (2004). Contributions of functional imaging to understanding parkinsonian symptoms. Curr. Opin. Neurobiol..

[b0120] Gu Q., Zhang H., Xuan M., Luo W., Huang P., Xia S., Zhang M. (2016). Automatic classification on multi-modal MRI data for diagnosis of the postural instability and gait difficulty subtype of Parkinson's disease. J. Parkinsons Dis..

[b0125] Guo X., Tinaz S., Dvornek N.C. (2022). Characterization of early stage Parkinson's disease from resting-state fMRI data using a long short-term memory network. Front Neuroimaging..

[b0130] Haaxma C.A., Bloem B.R., Borm G.F., Oyen W.J., Leenders K.L., Eshuis S., Booij J., Dluzen D.E., Horstink M.W. (2007). Gender differences in Parkinson's disease. J. Neurol. Neurosurg. Psychiatry.

[b0135] Haslinger B., Erhard P., Kämpfe N., Boecker H., Rummeny E., Schwaiger M., Conrad B., Ceballos-Baumann A.O. (2001). Event-related functional magnetic resonance imaging in Parkinson's disease before and after levodopa. Brain.

[b0140] He H., Liang L., Tang T., Luo J., Wang Y., Cui H. (2020). Progressive brain changes in parkinson’s disease: A meta-analysis of Structural Magnetic Resonance Imaging Studies. Brain Res..

[b0145] Herb J.N., Rane S., Isaacs D.A., Van Wouwe N., Roman O.C., Landman B.A., Dawant B.M., Hedera P., Zald D.H., Neimat J.S., Wylie S.A., Donahue M.J., Claassen D.O. (2016). Cortical Implications of Advancing Age and Disease Duration in Parkinson's Disease Patients with Postural Instability and Gait Dysfunction. J. Parkinsons Dis..

[b0150] Ho D., Imai K., King G., Stuart E. (2011). “MatchIt: Nonparametric Preprocessing for Parametric Causal Inference.” (version 4.5.5). J. Stat. Softw..

[b0155] https://www.ppmi-info.org (accessed 12 March 2023).

[b0160] Jahanshahi M., Jenkins I.H., Brown R.G., Marsden C.D., Passingham R.E., Brooks D.J. (1995). Self-initiated versus externally triggered movements. I. An investigation using measurement of regional cerebral blood flow with PET and movement-related potentials in normal and Parkinson's disease subjects. Brain.

[b0165] Jankovic J., McDermott M., Carter J., Gauthier S., Goetz C., Golbe L., Huber S., Koller W., Olanow C., Shoulson I. (1990). Variable expression of Parkinson's disease: a base-line analysis of the DATATOP cohort. The Parkinson Study Group. Neurology..

[b0175] Kang D.Z., Chen F.Y., Wang F.Y., Wu G.R., Liu Y., Wu G., Yu L.H., Lin Y.X., Lin Z.Y. (2015). Brain gray matter volume changes associated with motor symptoms in patients with Parkinson’s disease. Chin Neurosurg Jl..

[b0180] Kann S.J., Chang C., Manza P., Leung H.C. (2020). Akinetic rigid symptoms are associated with decline in a cortical motor network in Parkinson's disease. NPJ Parkinsons Dis..

[b0185] Kohat A.K., Ng S.Y.E., Wong A.S.Y., Chia N.S.Y., Choi X., Heng D.L., Li W., Ng H.L., Chua S.T., Neo S.X.M., Xu Z., Tay K.Y., Au W.L., Tan E.K., Tan L.C.S. (2021). Stability of MDS-UPDRS Motor Subtypes Over Three Years in Early Parkinson's Disease. Front. Neurol..

[b0190] Konno T., Deutschländer A., Heckman M.G., Ossi M., Vargas E.R., Strongosky A.J., van Gerpen J.A., Uitti R.J., Ross O.A., Wszolek Z.K. (2018). Comparison of clinical features among Parkinson's disease subtypes: A large retrospective study in a single center. J. Neurol. Sci..

[b0195] Li X., Xing Y., Martin-Bastida A., Piccini P., Auer D.P. (2017). Patterns of grey matter loss associated with motor subscores in early Parkinson's disease. Neuroimage Clin..

[b0200] Li J., Zhang Y., Huang Z., Jiang Y., Ren Z., Liu D., Zhang J., La Piana R., Chen Y. (2022). Cortical and subcortical morphological alterations in motor subtypes of Parkinson's disease. NPJ Parkinsons Dis..

[b0205] Lloyd B. Home [Internet]. GP2. 2022 [cited 2024 Oct 19]. Available from: https://gp2.org/.

[b0210] Marek K., Chowdhury S., Siderowf A., Lasch S., Coffey C.S., Caspell-Garcia C., Simuni T., Jennings D., Tanner C.M., Trojanowski J.Q., Shaw L.M., Seibyl J., Schuff N., Singleton A., Kieburtz K., Toga A.W., Mollenhauer B., Galasko D., Chahine L.M., Weintraub D., Foroud T., Tosun-Turgut D., Poston K., Arnedo V., Frasier M., Sherer T. (2018). Parkinson's Progression Markers Initiative. The Parkinson's progression markers initiative (PPMI) - establishing a PD biomarker cohort. Ann. Clin. Transl. Neurol..

[b0215] Matías-Guiu J.A., Cortés-Martínez A., Montero P., Pytel V., Moreno-Ramos T., Jorquera M., Yus M., Arrazola J., Matías-Guiu J. (2018). Identification of Cortical and Subcortical Correlates of Cognitive Performance in Multiple Sclerosis Using Voxel-Based Morphometry. Front. Neurol..

[b0220] Mestre T.A., Eberly S., Tanner C., Grimes D., Lang A.E., Oakes D., Marras C. (2018). Reproducibility of data-driven Parkinson's disease subtypes for clinical research. Parkinsonism Relat. Disord..

[b0225] Mestre T.A., Fereshtehnejad S.M., Berg D., Bohnen N.I., Dujardin K., Erro R., Espay A.J., Halliday G., van Hilten J.J., Hu M.T., Jeon B., Klein C., Leentjens A.F.G., Marinus J., Mollenhauer B., Postuma R., Rajalingam R., Rodríguez-Violante M., Simuni T., Surmeier D.J., Weintraub D., McDermott M.P., Lawton M., Marras C. (2021). Parkinson's Disease Subtypes: Critical Appraisal and Recommendations. J. Parkinsons Dis..

[b0230] Mitchell T., Lehéricy S., Chiu S.Y., Strafella A.P., Stoessl A.J., Vaillancourt D.E. (2021). Emerging Neuroimaging Biomarkers Across Disease Stage in Parkinson Disease: A Review. JAMA Neurol..

[b0235] Nachev P., Kennard C., Husain M. (2008). Functional role of the supplementary and pre-supplementary motor areas. Nat. Rev. Neurosci..

[b0240] Nassif J, Martin A, Chaluvadi L, Schammel C, Absher J. Novel Analysis to Compare Parkinson’s Disease Motor Subtypes. Poster presented at Discover USC; 19 April 2024; University of South Carolina - Columbia, SC.

[b0245] Oltra J., Segura B., Strafella A.P., van Eimeren T., Ibarretxe-Bilbao N., Diez-Cirarda M. (2024). A multi-site study on sex differences in cortical thickness in non-demented Parkinson’s disease. Npj Parkinson s Disease..

[b0250] Oswal A., Cao C., Yeh C.H., Neumann W.J., Gratwicke J., Akram H., Horn A., Li D., Zhan S., Zhang C., Wang Q., Zrinzo L., Foltynie T., Limousin P., Bogacz R., Sun B., Husain M., Brown P., Litvak V. (2021). Neural signatures of hyperdirect pathway activity in Parkinson's disease. Nat. Commun..

[b0255] Peng B., Wang S., Zhou Z., Liu Y., Tong B., Zhang T., Dai Y. (2017). A multilevel-ROI-features-based machine learning method for detection of morphometric biomarkers in Parkinson's disease. Neurosci. Lett..

[b0260] Pereira J.B., Svenningsson P., Weintraub D., Brønnick K., Lebedev A., Westman E., Aarsland D. (2014 3;82(22):2017–25.). Initial cognitive decline is associated with cortical thinning in early Parkinson disease. Neurology.

[b0265] Playford E.D., Jenkins I.H., Passingham R.E., Nutt J., Frackowiak R.S., Brooks D.J. (1992). Impaired mesial frontal and putamen activation in Parkinson's disease: a positron emission tomography study. Ann. Neurol..

[b0270] Poewe W., Seppi K., Tanner C.M., Halliday G.M., Brundin P., Volkmann J., Schrag A.E., Lang A.E. (2017). Parkinson Disease. Nat Rev Dis Primers..

[b0275] Prashanth R., Dutta Roy S., Mandal P.K., Ghosh S. (2016). High-Accuracy Detection of Early Parkinson's Disease through Multimodal Features and Machine Learning. Int. J. Med. Inf..

[b0280] Pringsheim T., Jette N., Frolkis A., Steeves T.D. (2014). The prevalence of Parkinson's disease: a systematic review and meta-analysis. Mov. Disord..

[b0285] Rahimpour S., Rajkumar S., Hallett M. (2022 Jan). The Supplementary Motor Complex in Parkinson's Disease. J Mov Disord..

[b0290] Rajput A.H., Voll A., Rajput M.L., Robinson C.A., Rajput A. (2009). Course in Parkinson disease subtypes: A 39-year clinicopathologic study. Neurology.

[b0295] Ren J., Hua P., Li Y., Pan C., Yan L., Yu C., Zhang L., Xu P., Zhang M., Liu W. (2020). Comparison of Three Motor Subtype Classifications in de novo Parkinson's Disease Patients. Front. Neurol..

[b0300] Renton A.I., Dao T.T., Johnstone T., Civier O., Sullivan R.P., White D.J., Narayanan A., Bollmann S. (2024). Neurodesk: an accessible, flexible and portable data analysis environment for reproducible neuroimaging. Nat. Methods.

[b0305] Rosenberg-Katz K., Herman T., Jacob Y., Giladi N., Hendler T., Hausdorff J.M. (2013). Gray matter atrophy distinguishes between Parkinson disease motor subtypes. Neurology.

[b0310] Rosenberg-Katz K., Herman T., Jacob Y., Kliper E., Giladi N., Hausdorff J.M. (2016). Subcortical Volumes Differ in Parkinson's Disease Motor Subtypes: New Insights into the Pathophysiology of Disparate Symptoms. Front. Hum. Neurosci..

[b0315] Russillo M.C., Andreozzi V., Erro R., Picillo M., Amboni M., Cuoco S., Barone P., Pellecchia M.T. (2022). Sex Differences in Parkinson's Disease: From Bench to Bedside. Brain Sci..

[b0320] Sadler C.M., Kami A.T., Nantel J., Carlsen A.N. (2021). Transcranial direct current stimulation of supplementary motor area improves upper limb kinematics in Parkinson's disease. Clin. Neurophysiol..

[b0325] Shima K., Tanji J. (1998). Both supplementary and presupplementary motor areas are crucial for the temporal organization of multiple movements. J. Neurophysiol..

[b0330] Sieber B., Landis S.C., Koroshetz W.J., Bateman R.J., Siderowf A., Galpern W.R. (2014). Prioritized research recommendations from the National Institute of Neurological Disorders and Stroke *Parkinson’s Disease 2014 conference*. Ann. Neurol..

[b0335] Simuni T., Caspell-Garcia C., Coffey C., Lasch S., Tanner C., Marek K., Investigators P.P.M.I. (2016). How stable are Parkinson's disease subtypes in de novo patients: Analysis of the PPMI cohort?. Parkinsonism Relat. Disord..

[b0340] Stebbins G.T., Goetz C.G., Burn D.J., Jankovic J., Khoo T.K., Tilley B.C. (2013). How to identify tremor dominant and postural instability/gait difficulty groups with the movement disorder society unified Parkinson’s disease rating scale: Comparison with the unified Parkinson’s disease rating scale: PIGD and The MDS-UPDRS. Mov. Disord..

[b0345] Unda S.R., Marciano S., Milner T.A., Marongiu R. (2022). State-of-the-art review of the clinical research on menopause and hormone replacement therapy association with Parkinson's disease: What meta-analysis studies cannot tell us. Front. Aging Neurosci..

[b0350] Uribe C., Segura B., Baggio H.C., Abos A., Garcia-Diaz A.I., Campabadal A., Marti M.J., Valldeoriola F., Compta Y., Tolosa E., Junque C. (2018). Cortical atrophy patterns in early Parkinson's disease patients using hierarchical cluster analysis. Parkinsonism Relat. Disord..

[b0355] Willis A.W., Roberts E., Beck J.C., Fiske B., Ross W., Savica R., Van Den Eeden S.K., Tanner C.M., Marras C. (2022). Parkinson’s Foundation P4 Group. Incidence of Parkinson disease in North America. NPJ Parkinsons Dis..

[b0360] Wu T., Wang L., Chen Y., Zhao C., Li K., Chan P. (2009). Changes of functional connectivity of the motor network in the resting state in Parkinson's disease. Neurosci. Lett..

[b0365] Wu T., Wang J., Wang C., Hallett M., Zang Y., Wu X., Chan P. (2012). Basal ganglia circuit changes in Parkinson's disease patients. Neurosci. Lett..

[b0370] Yadav S.K., Kathiresan N., Mohan S., Vasileiou G., Singh A., Kaura D., Melhem E.R., Gupta R.K., Wang E., Marincola F.M., Borthakur A., Haris M. (2016). Gender-based analysis of cortical thickness and structural connectivity in Parkinson's disease. J. Neurol..

[b0375] Zeighami Y., Fereshtehnejad S.M., Dadar M., Collins D.L., Postuma R.B., Dagher A. (2019). Assessment of a prognostic MRI biomarker in early de novo Parkinson's disease. Neuroimage Clin..

[b0380] Zetusky W.J., Jankovic J., Pirozzolo F.J. (1985). The heterogeneity of Parkinson's disease: clinical and prognostic implications. Neurology.

[b0385] Zhang J. (2022). Mining imaging and clinical data with machine learning approaches for the diagnosis and early detection of Parkinson's disease. NPJ Parkinsons Dis..

[b0390] Zheng J.H., Sun W.H., Ma J.J., Wang Z.D., Chang Q.Q., Dong L.R., Shi X.X., Li M.J., Gu Q., Chen S.Y., Li D.S. (2022). Differences in neuroanatomy and functional connectivity between motor subtypes of Parkinson's disease. Front. Neurosci..

